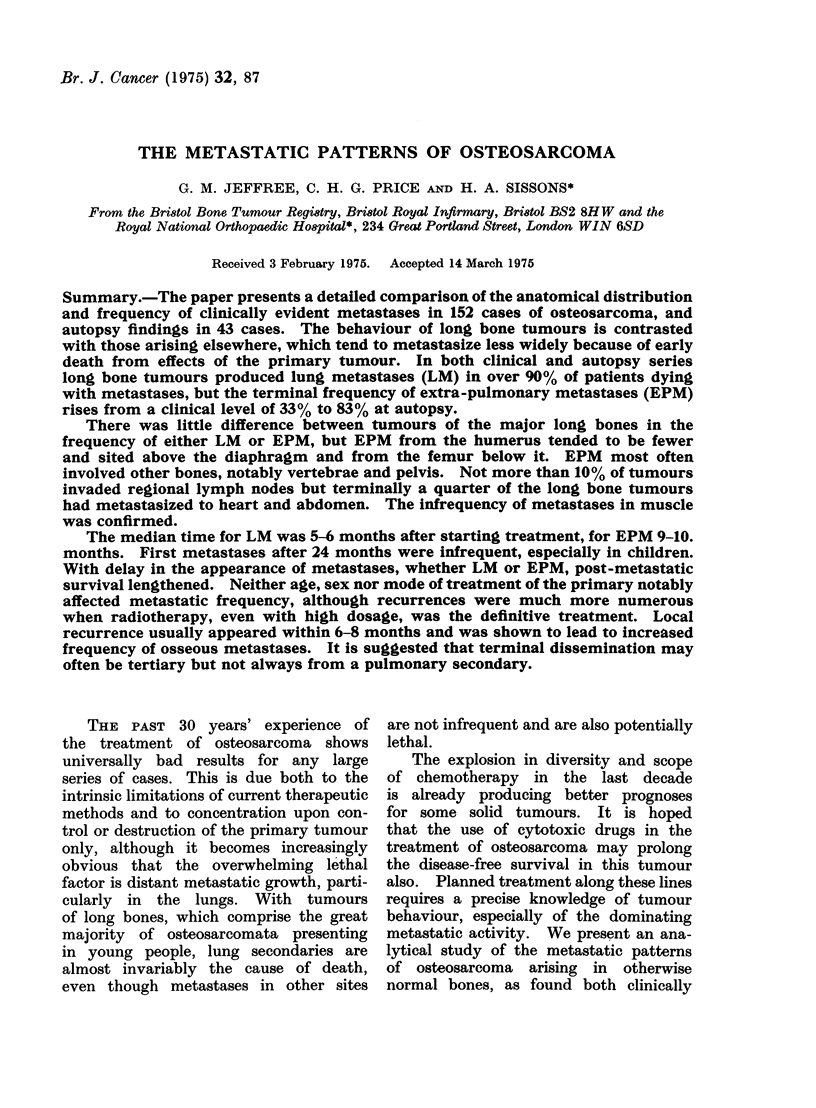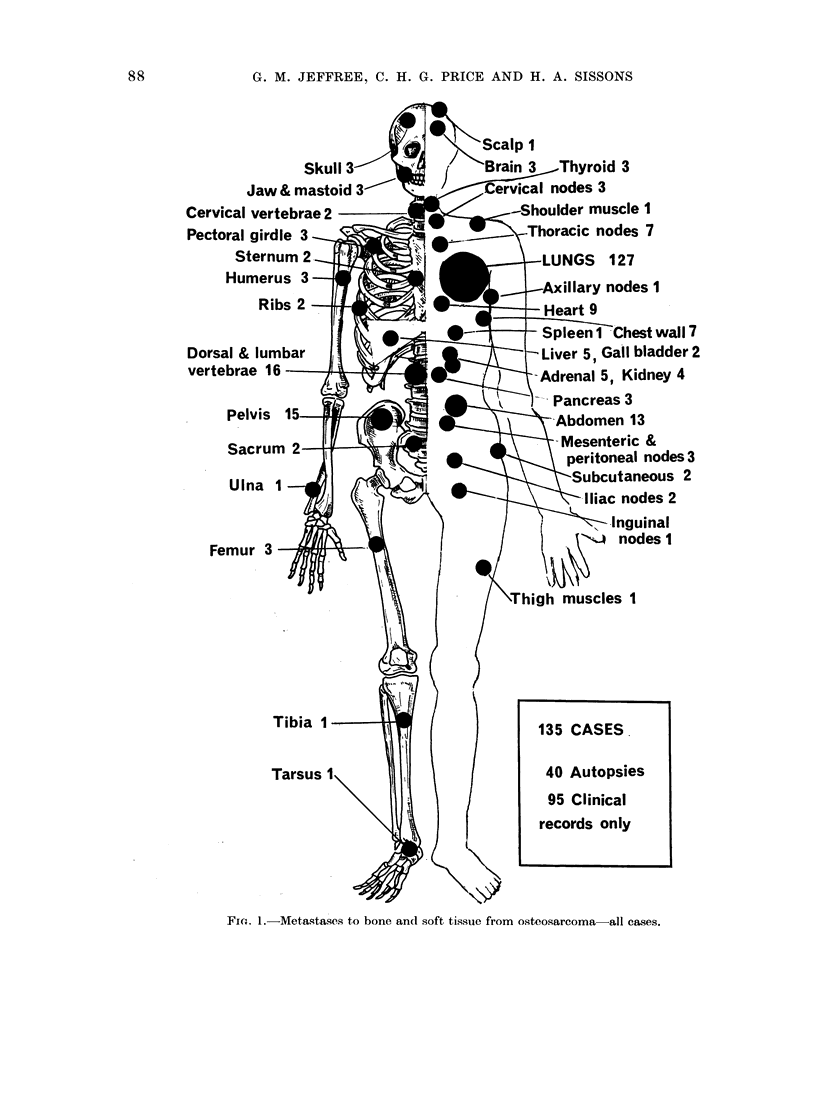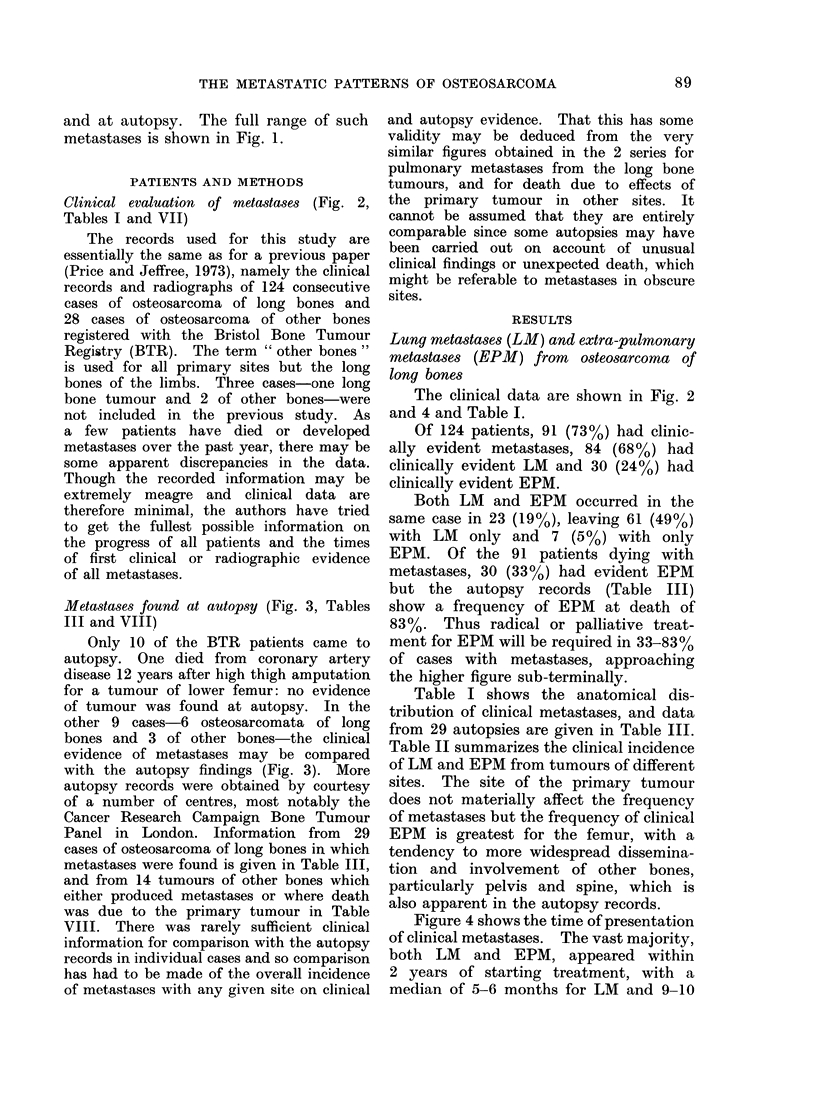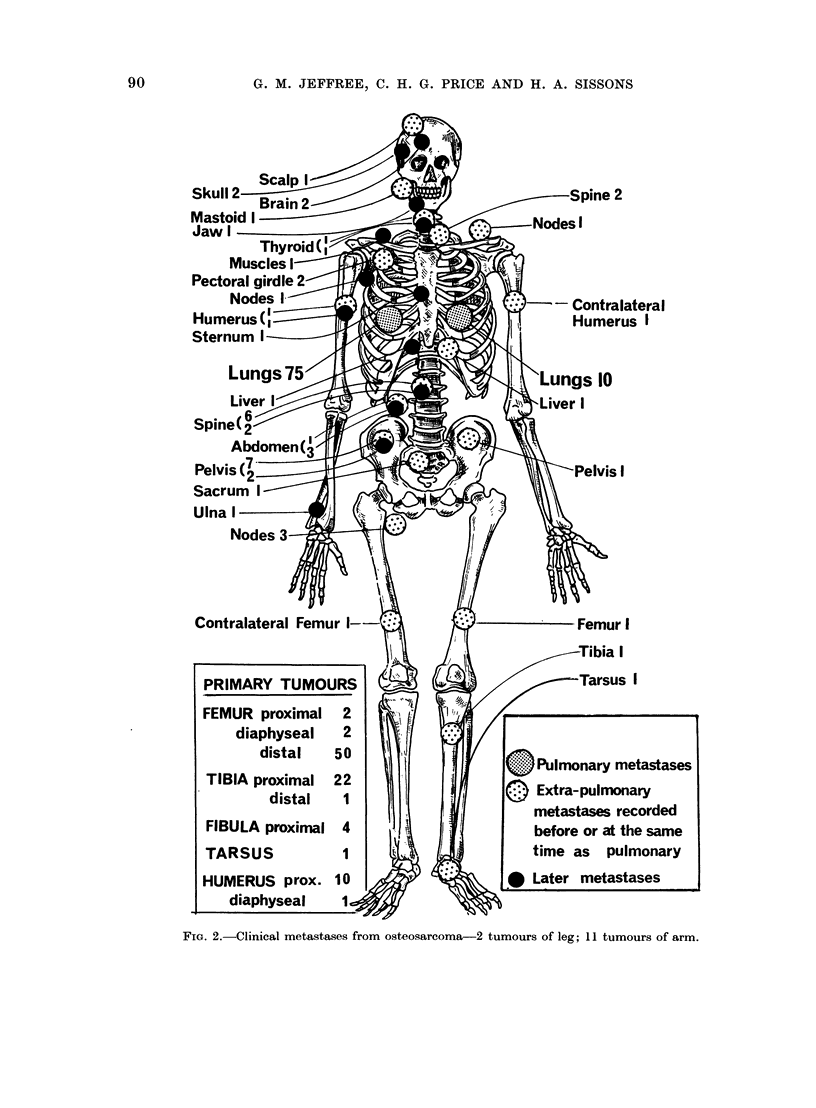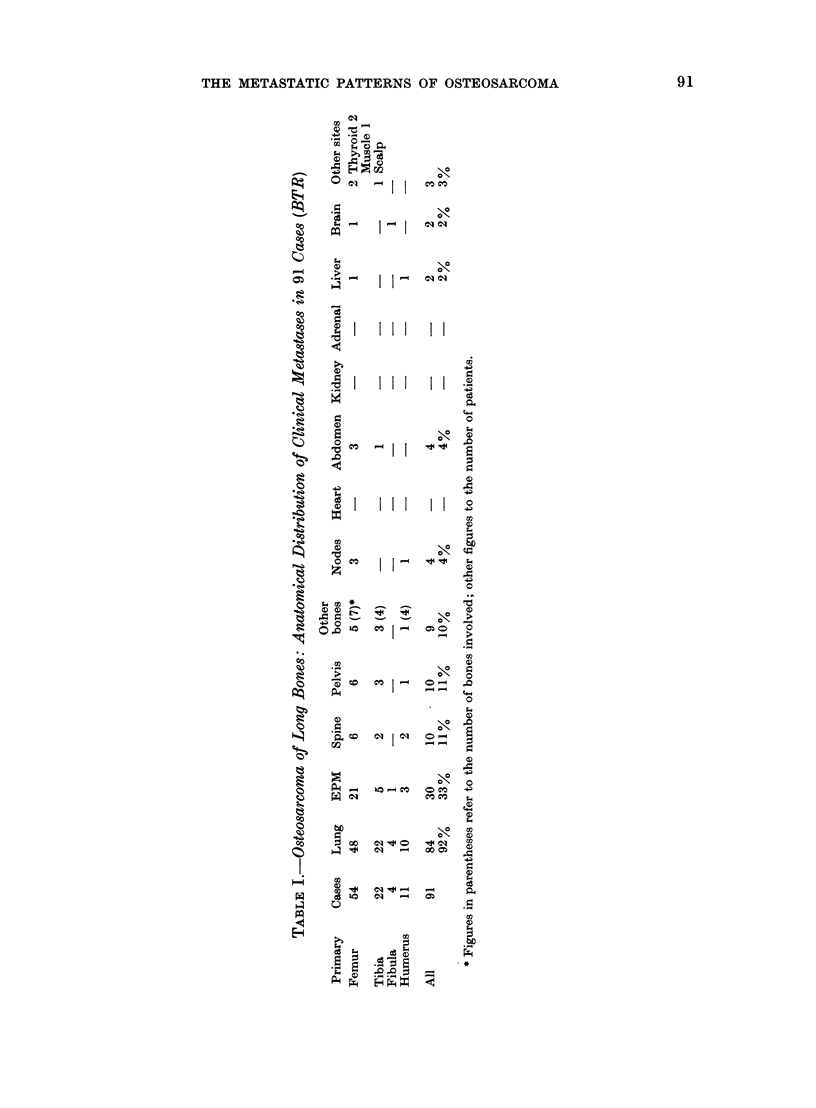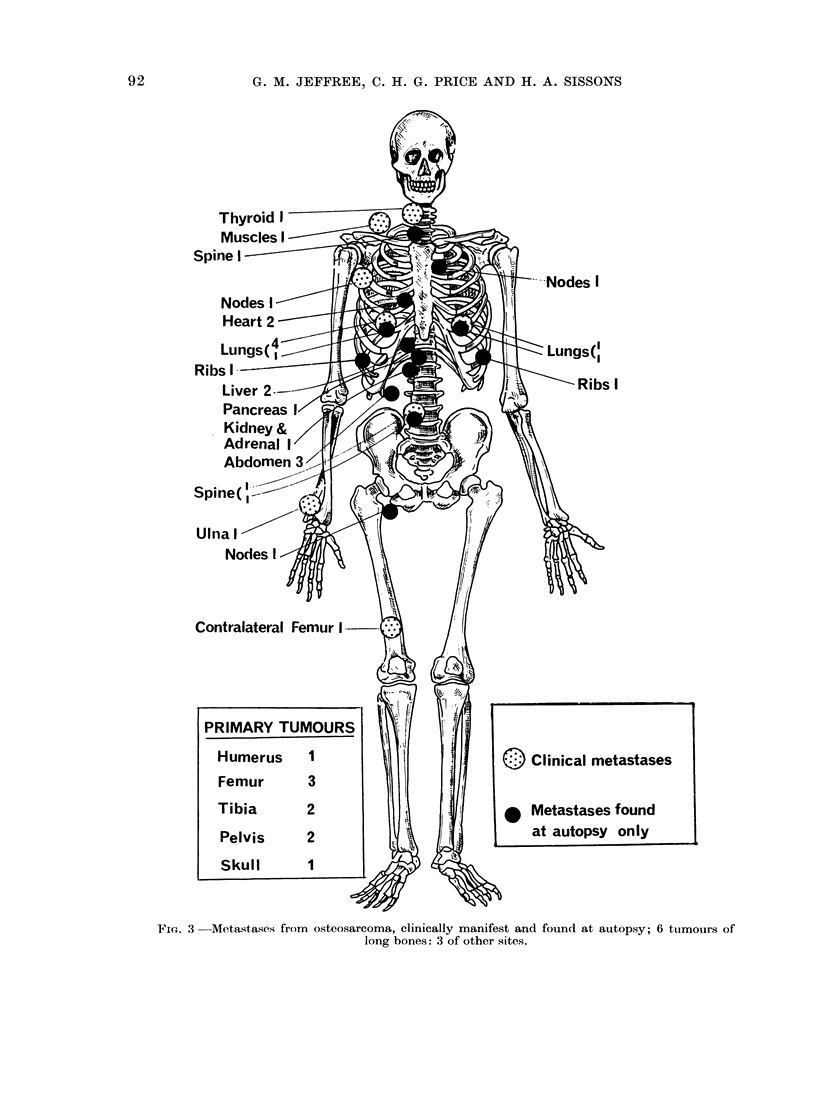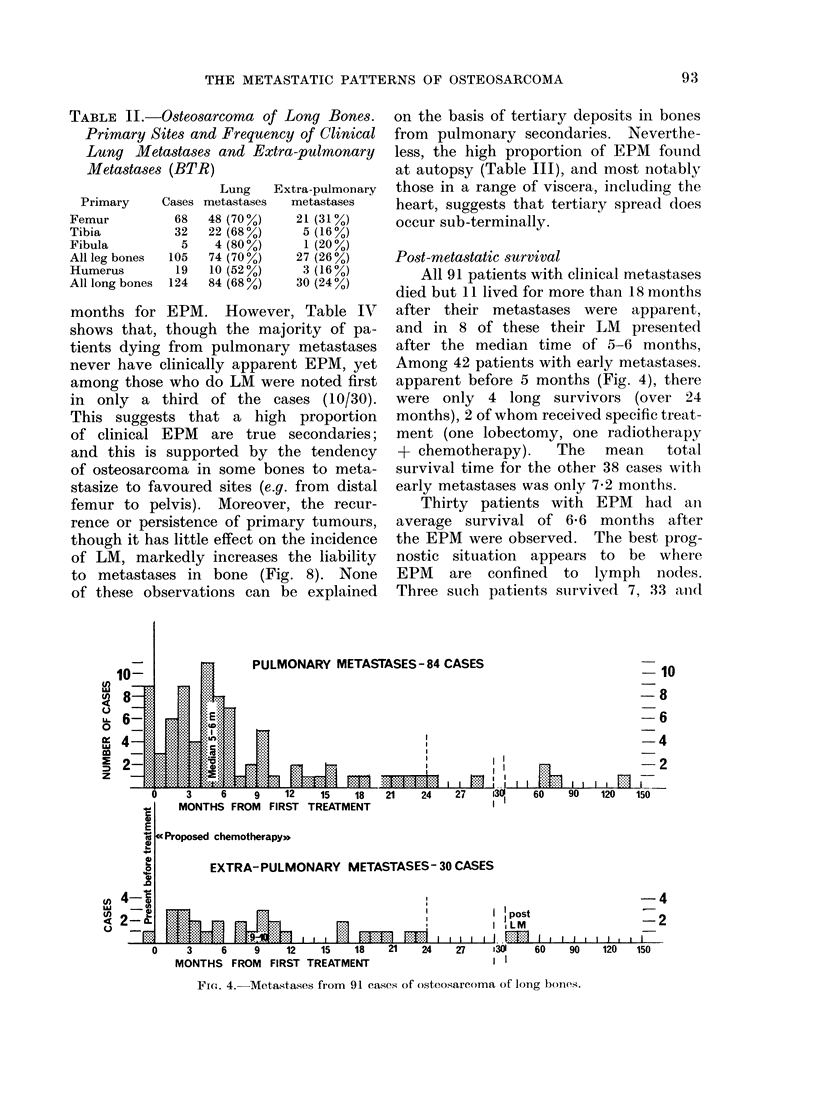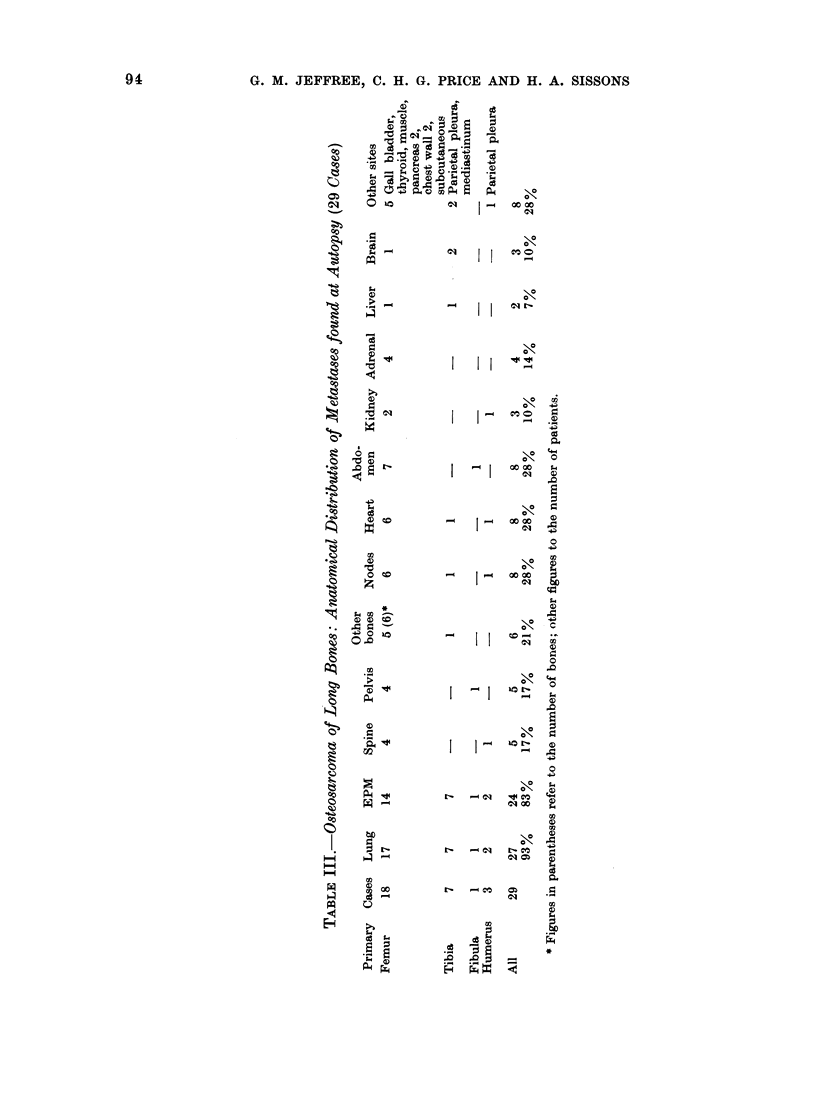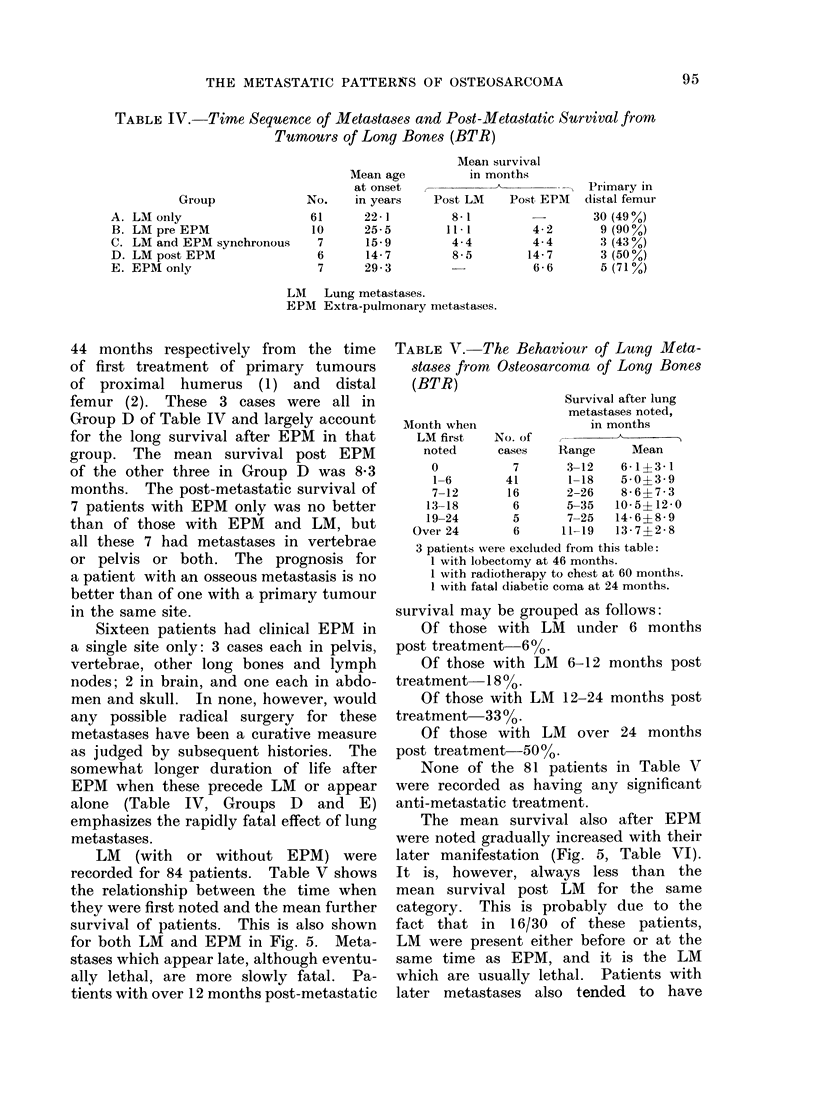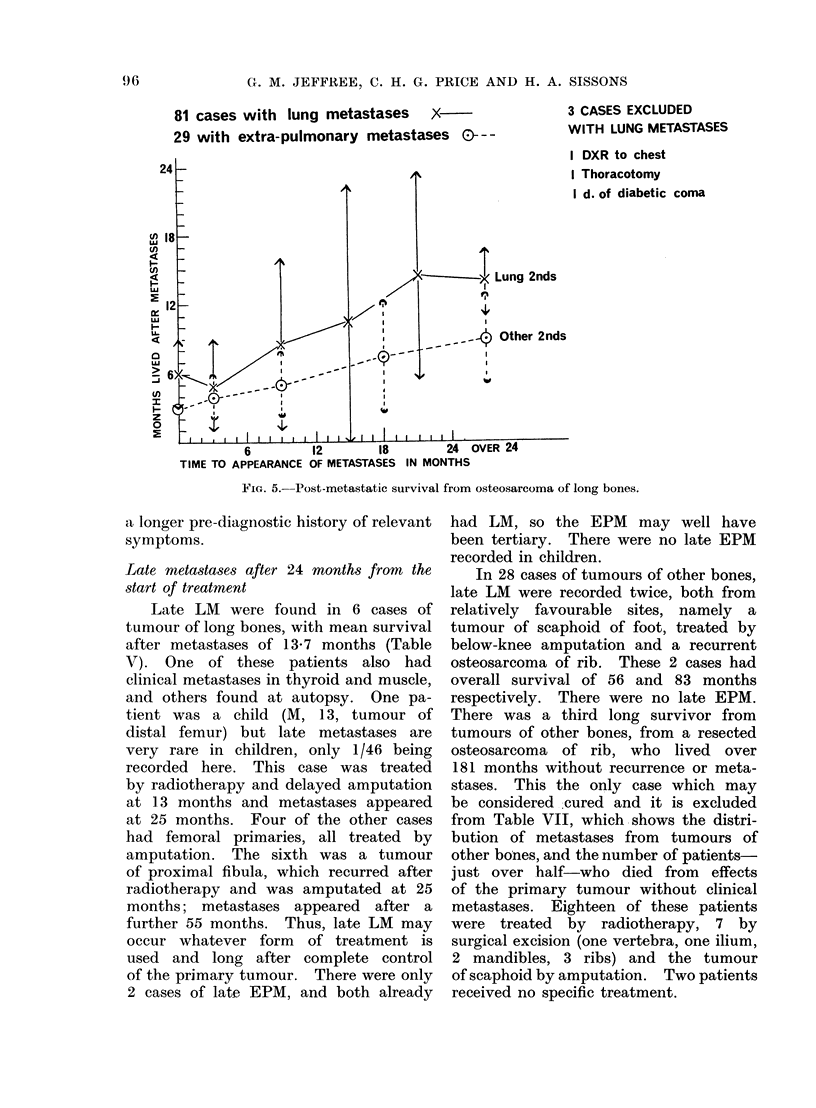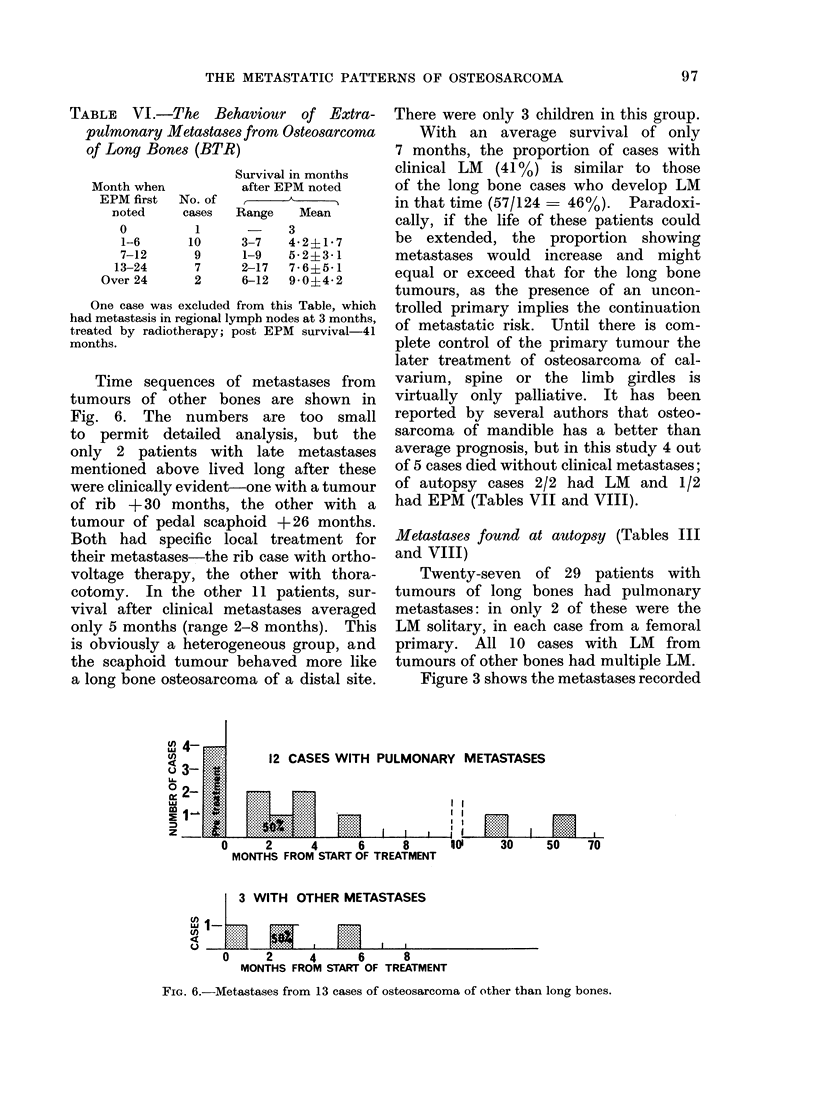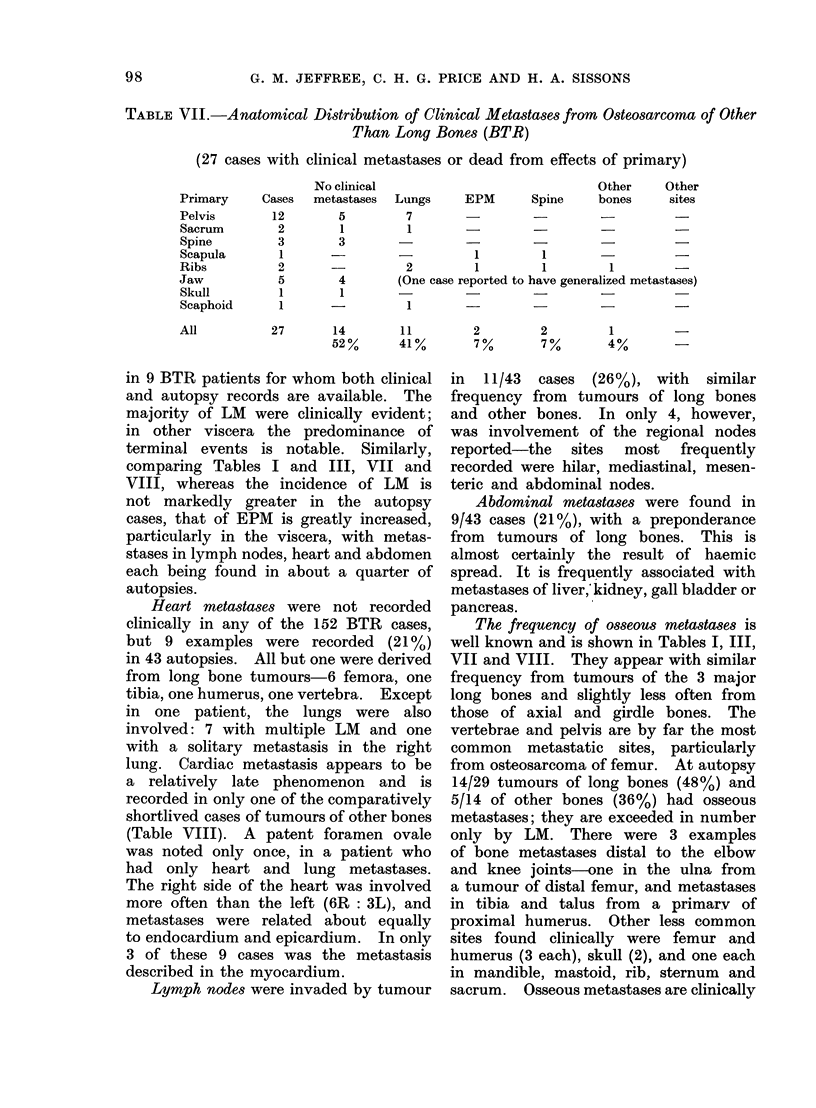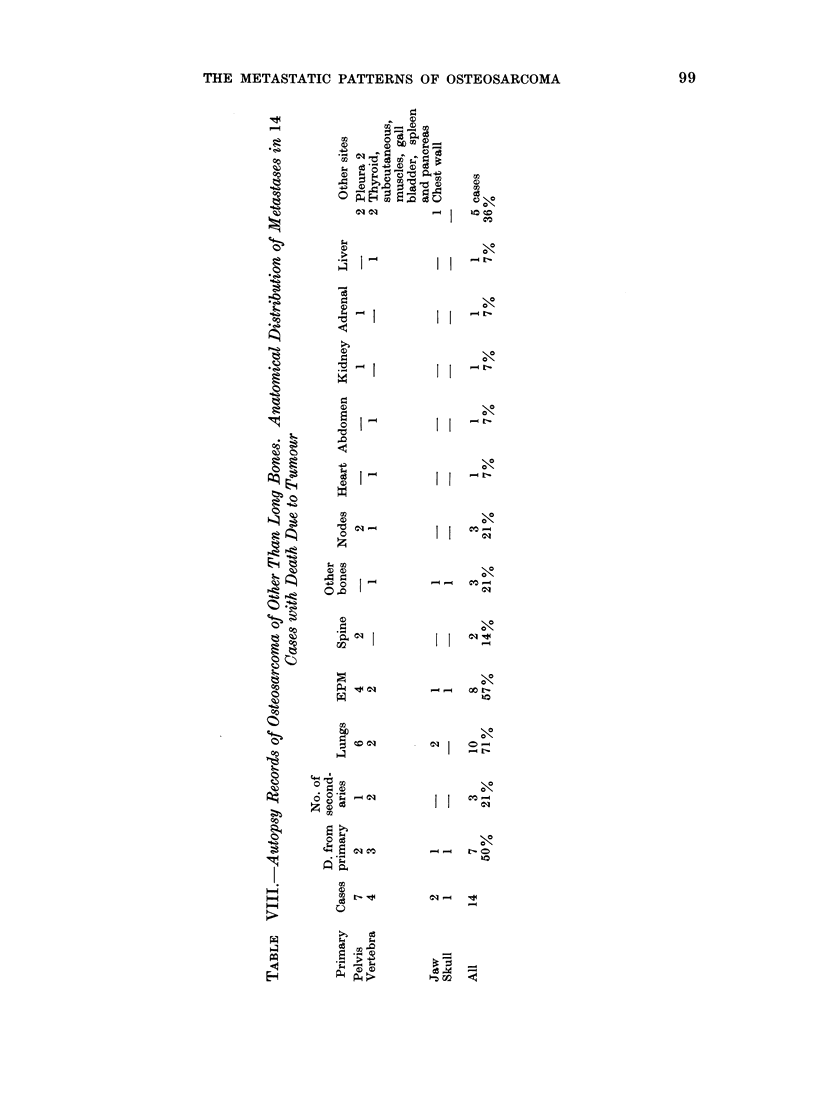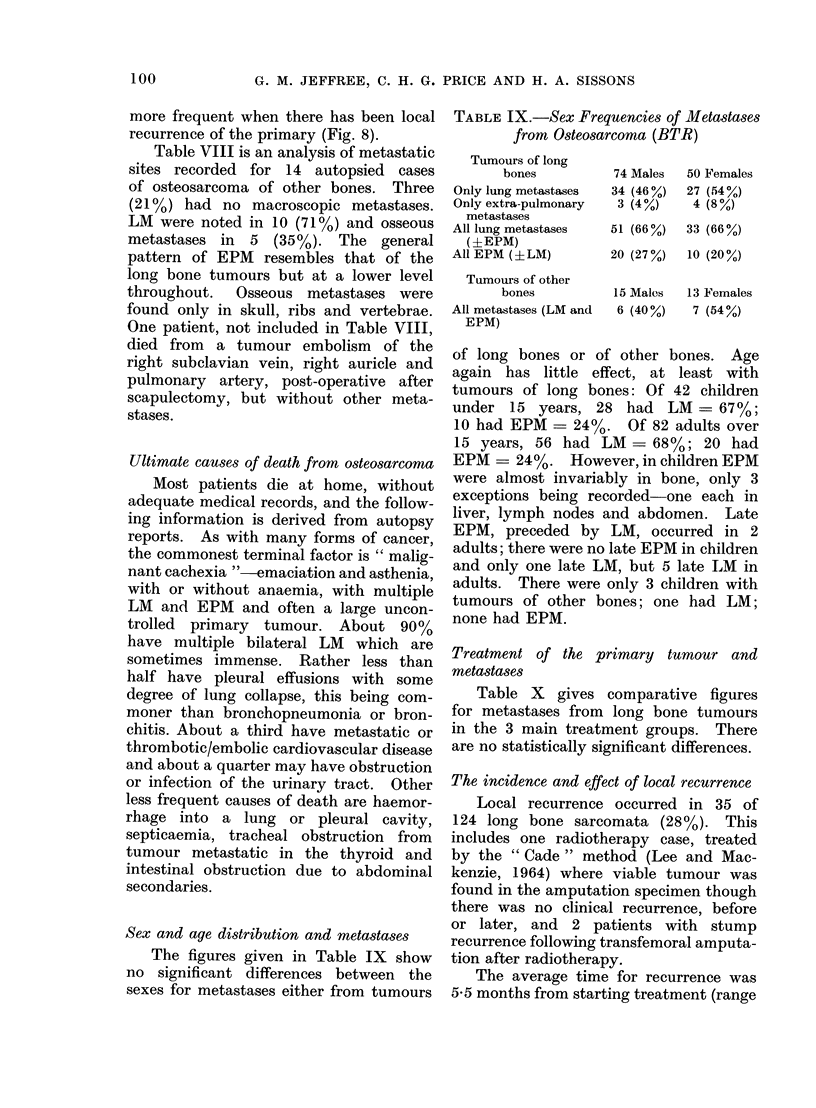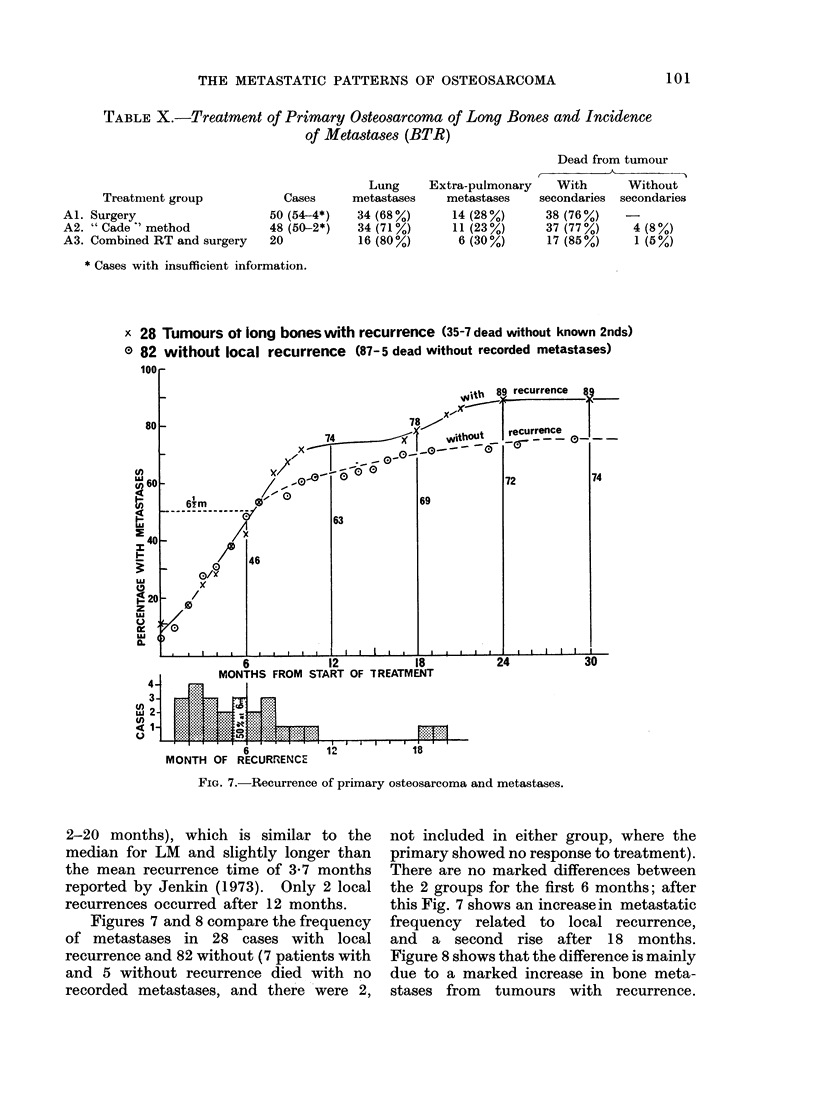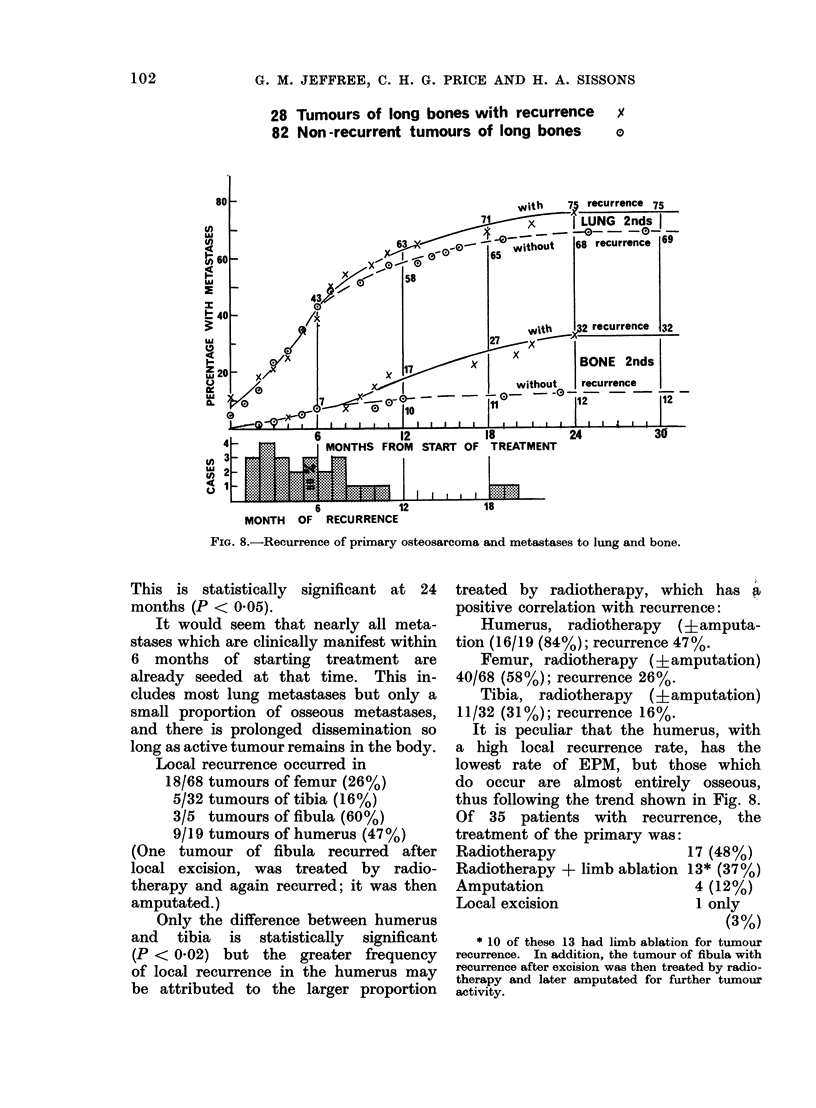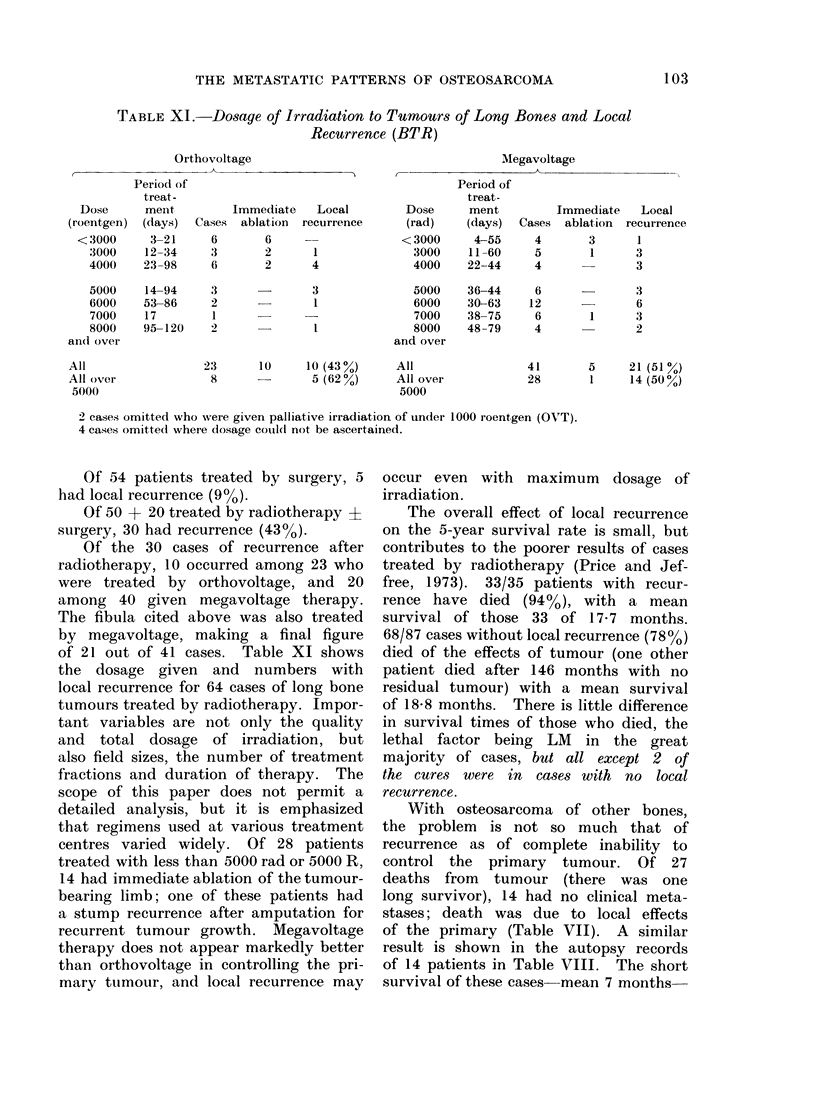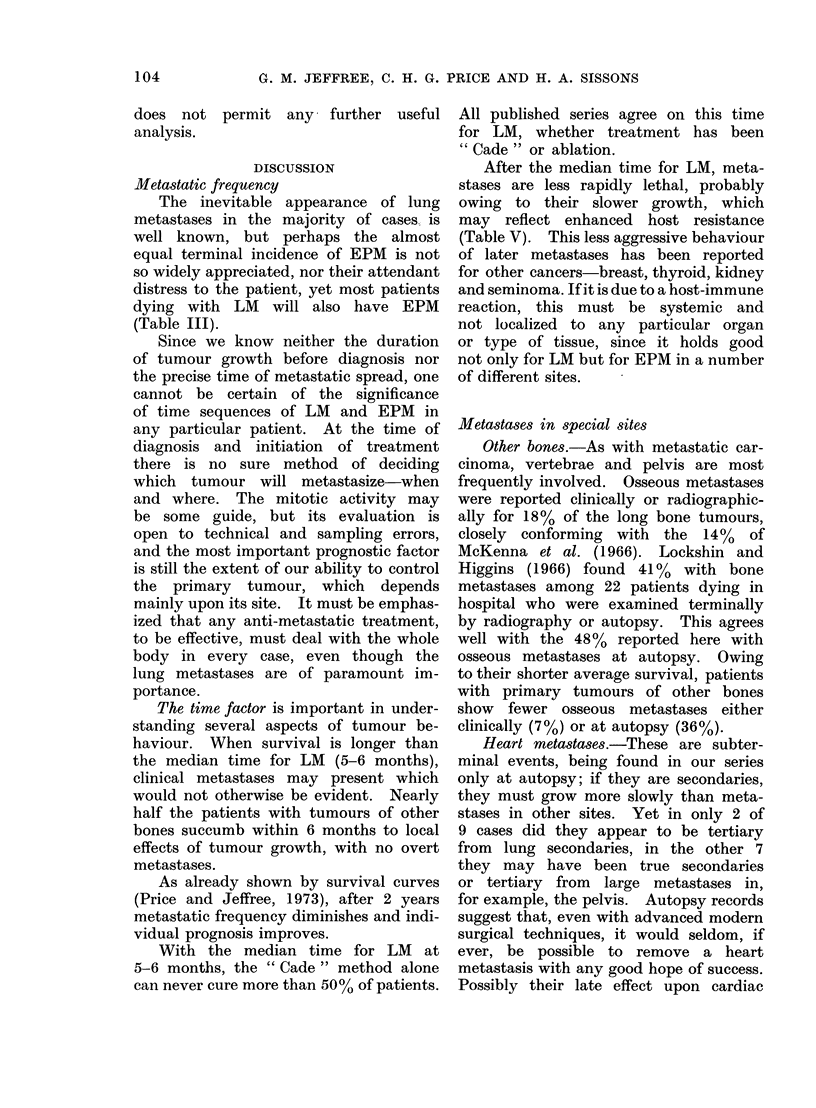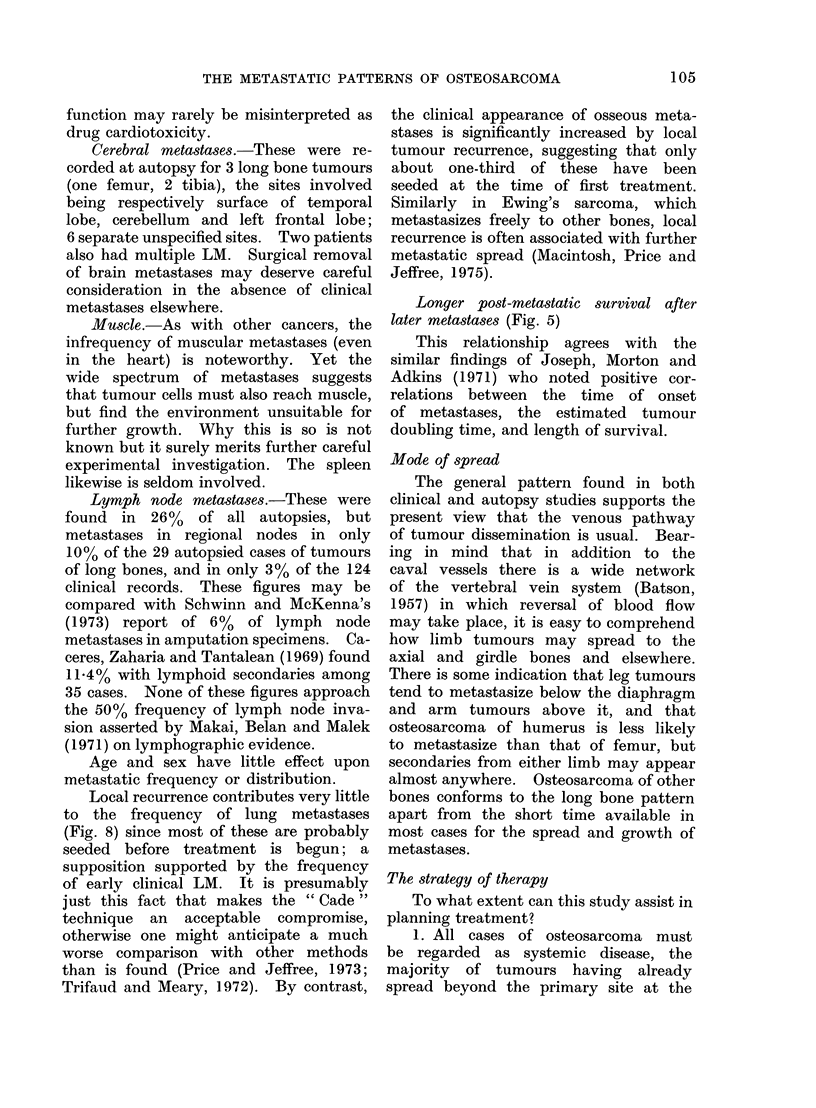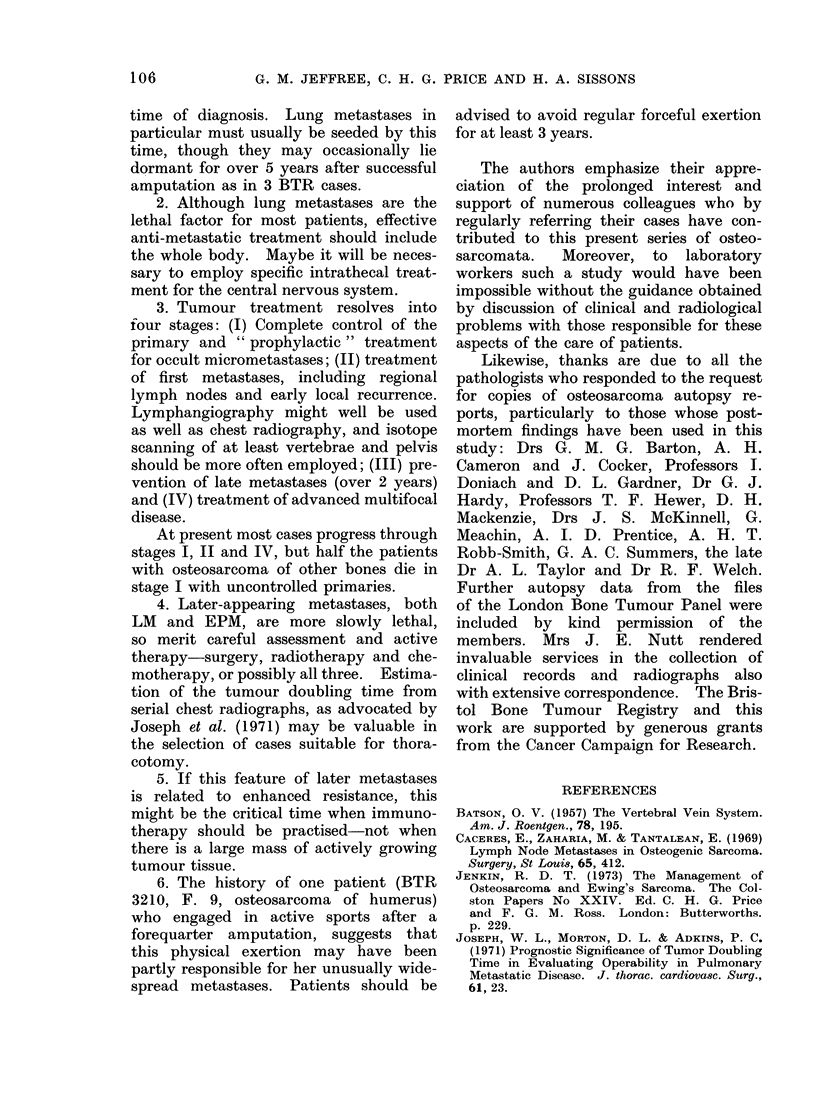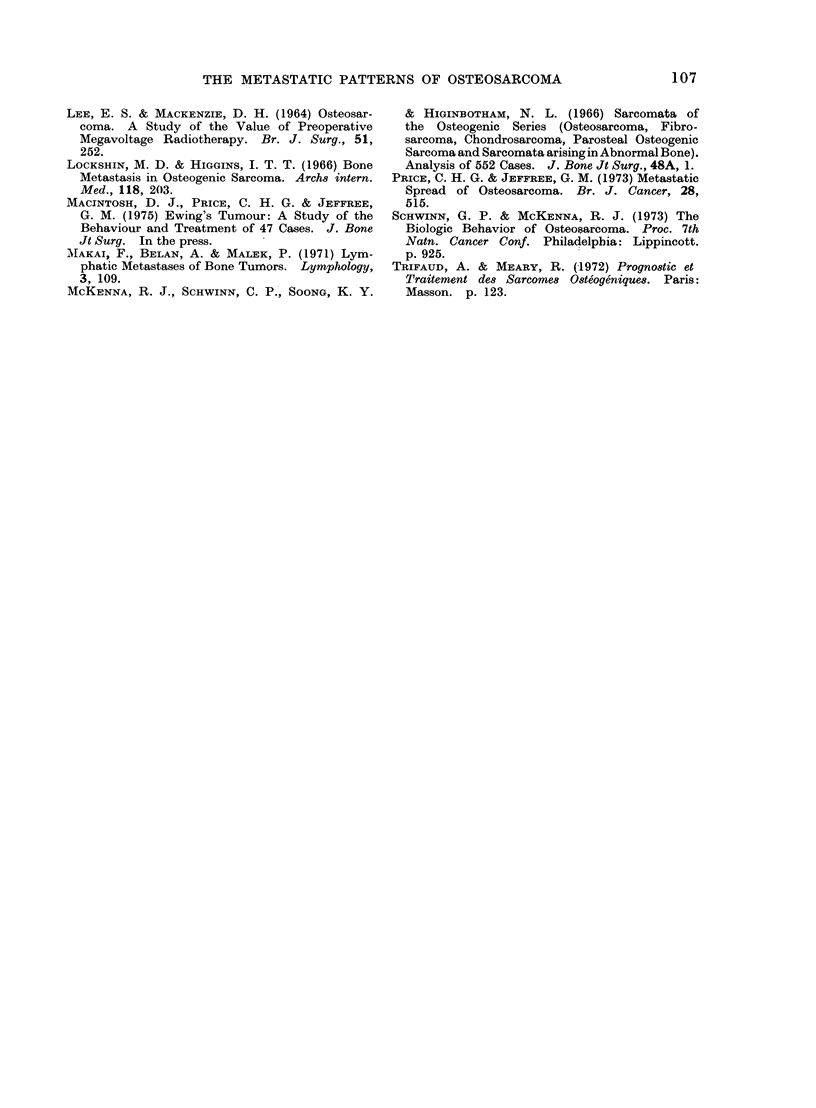# The metastatic patterns of osteosarcoma.

**DOI:** 10.1038/bjc.1975.136

**Published:** 1975-07

**Authors:** G. M. Jeffree, C. H. Price, H. A. Sissons

## Abstract

The paper presents a detailed comparison of the anatomical distribution and frequency of clinically evident metastases in 152 cases of osteosarcoma, and autopsy findings in 43 cases. The behaviour of long bone tumours is contrasted with those arising elsewhere, which tend to metastasize less widely because of early death from effects of the primary tumour. In both clinical and autopsy series long bone tumours produced lung metastases (LM) in over 90% of patients dying with metastases, but the terminal frequency of extra-pulmonary metastases (EPM) rises from a clinical level of 33% to 83% at autopsy. There was little difference between tumours of the major long bones in the frequency of either LM or EPM, but EPM from the humerus tended to be fewer and sited above the diaphragm and from the femur below it. EPM most often involved other bones, notably vertebrae and pelvis. Not more than 10% of tumours invaded regional lymph nodes but terminally a quarter of the long bone tumours had metastasized to heart and abdomen. The infrequency of metastases in muscle was confirmed. The median time for LM was 5-6 months after starting treatment, for EPM 9-10. months. First metastases after 24 months were infrequent, especially in children. With delay in the appearance of metastases, whether LM or EPM, post-metastatic survival lengthened. Neither age, sex nor mode of treatment of the primary notably affected metastatic frequency, although recurrences were much more numerous when radiotherapy, even with high dosage, was the definitive treatment. Local recurrence usually appeared within 6-8 months and was shown to lead to increased frequency of osseous metastases. It is suggested that terminal dissemination may often be tertiary but not always from a pulmonary secondary.


*Br. J. Cancer* (1975) **32**, 87

### THE METASTATIC PATTERNS OF OSTEOSARCOMA

G. M. JEFFREE, C. H. G. PRICE AND H. A. SISSONS*

*From the Bristol Bone Tumour Registry, Bristol Royal Infirmary, Bristol BS2 8HW and the Royal National Orthopaedic Hospital*, 234 Great Portland Street, London W1N 6SD*

Received 3 February 1975. Accepted 14 March 1975

**Summary.—The paper presents a detailed comparison of the anatomical distribution and frequency of clinically evident metastases in 152 cases of osteosarcoma, and autopsy findings in 43 cases. The behaviour of long bone tumours is contrasted with those arising elsewhere, which tend to metastasize less widely because of early death from effects of the primary tumour. In both clinical and autopsy series long bone tumours produced lung metastases (LM) in over 90% of patients dying with metastases, but the terminal frequency of extra-pulmonary metastases (EPM) rises from a clinical level of 33% to 83% at autopsy.**

**There was little difference between tumours of the major long bones in the frequency of either LM or EPM, but EPM from the humerus tended to be fewer and sited above the diaphragm and from the femur below it. EPM most often involved other bones, notably vertebrae and pelvis. Not more than 10% of tumours invaded regional lymph nodes but terminally a quarter of the long bone tumours had metastasized to heart and abdomen. The infrequency of metastases in muscle was confirmed.**

**The median time for LM was 5–6 months after starting treatment, for EPM 9–10. months. First metastases after 24 months were infrequent, especially in children. With delay in the appearance of metastases, whether LM or EPM, post-metastatic survival lengthened. Neither age, sex nor mode of treatment of the primary notably affected metastatic frequency, although recurrences were much more numerous when radiotherapy, even with high dosage, was the definitive treatment. Local recurrence usually appeared within 6–8 months and was shown to lead to increased frequency of osseous metastases. It is suggested that terminal dissemination may often be tertiary but not always from a pulmonary secondary.**

THE PAST 30 years' experience of the treatment of osteosarcoma shows universally bad results for any large series of cases. This is due both to the intrinsic limitations of current therapeutic methods and to concentration upon control or destruction of the primary tumour only, although it becomes increasingly obvious that the overwhelming lethal factor is distant metastatic growth, particularly in the lungs. With tumours of long bones, which comprise the great majority of osteosarcomata presenting in young people, lung secondaries are almost invariably the cause of death, even though metastases in other sites are not infrequent and are also potentially lethal.

The explosion in diversity and scope of chemotherapy in the last decade is already producing better prognoses for some solid tumours. It is hoped that the use of cytotoxic drugs in the treatment of osteosarcoma may prolong the disease-free survival in this tumour also. Planned treatment along these lines requires a precise knowledge of tumour behaviour, especially of the dominating metastatic activity. We present an analytical study of the metastatic patterns of osteosarcoma arising in otherwise normal bones, as found both clinically

FIG. 1.—Metastases to bone and soft tissue from osteosarcoma—all cases.

and at autopsy. The full range of such metastases is shown in Fig. 1.

### PATIENTS AND METHODS

*Clinical evaluation of metastases* (Fig. 2, Tables I and VII)

The records used for this study are essentially the same as for a previous paper (Price and Jeffree, 1973), namely the clinical records and radiographs of 124 consecutive cases of osteosarcoma of long bones and 28 cases of osteosarcoma of other bones registered with the Bristol Bone Tumour Registry (BTR). The term " other bones " is used for all primary sites but the long bones of the limbs. Three cases—one long bone tumour and 2 of other bones—were not included in the previous study. As a few patients have died or developed metastases over the past year, there may be some apparent discrepancies in the data. Though the recorded information may be extremely meagre and clinical data are therefore minimal, the authors have tried to get the fullest possible information on the progress of all patients and the times of first clinical or radiographic evidence of all metastases.

*Metastases found at autopsy* (Fig. 3, Tables III and VIII)

Only 10 of the BTR patients came to autopsy. One died from coronary artery disease 12 years after high thigh amputation for a tumour of lower femur: no evidence of tumour was found at autopsy. In the other 9 cases—6 osteosarcomata of long bones and 3 of other bones—the clinical evidence of metastases may be compared with the autopsy findings (Fig. 3). More autopsy records were obtained by courtesy of a number of centres, most notably the Cancer Research Campaign Bone Tumour Panel in London. Information from 29 cases of osteosarcoma of long bones in which metastases were found is given in Table III, and from 14 tumours of other bones which either produced metastases or where death was due to the primary tumour in Table VIII. There was rarely sufficient clinical information for comparison with the autopsy records in individual cases and so comparison has had to be made of the overall incidence of metastases with any given site on clinical and autopsy evidence. That this has some validity may be deduced from the very similar figures obtained in the 2 series for pulmonary metastases from the long bone tumours, and for death due to effects of the primary tumour in other sites. It cannot be assumed that they are entirely comparable since some autopsies may have been carried out on account of unusual clinical findings or unexpected death, which might be referable to metastases in obscure sites.

### RESULTS

*Lung metastases (LM) and extra-pulmonary metastases (EPM) from osteosarcoma of long bones*

The clinical data are shown in Fig. 2 and 4 and Table I.

Of 124 patients, 91 (73%) had clinically evident metastases, 84 (68%) had clinically evident LM and 30 (24%) had clinically evident EPM.

Both LM and EPM occurred in the same case in 23 (19%), leaving 61 (49%) with LM only and 7 (5%) with only EPM. Of the 91 patients dying with metastases, 30 (33%) had evident EPM but the autopsy records (Table III) show a frequency of EPM at death of 83%. Thus radical or palliative treatment for EPM will be required in 33–83% of cases with metastases, approaching the higher figure sub-terminally.

Table I shows the anatomical distribution of clinical metastases, and data from 29 autopsies are given in Table III. Table II summarizes the clinical incidence of LM and EPM from tumours of different sites. The site of the primary tumour does not materially affect the frequency of metastases but the frequency of clinical EPM is greatest for the femur, with a tendency to more widespread dissemination and involvement of other bones, particularly pelvis and spine, which is also apparent in the autopsy records.

Figure 4 shows the time of presentation of clinical metastases. The vast majority, both LM and EPM, appeared within 2 years of starting treatment, with a median of 5–6 months for LM and 9–10

FIG. 2.—Clinical metastases from osteosarcoma—2 tumours of leg; 11 tumours of arm.

TABLE I.—*Osteosarcoma of Long Bones: Anatomical Distribution of Clinical Metastases in* 91 *Cases* (*BTR*)

| Primary | Cases | Lung | EPM | Spine | Pelvis | Other bones | Nodes | Heart | Abdomen | Kidney | Adrenal | Liver | Brain | Other sites |
|---|---|---|---|---|---|---|---|---|---|---|---|---|---|---|
| Femur | 54 | 48 | 21 | 6 | 6 | 5 (7)* | 3 | — | 3 | — | — | 1 | 1 | 2 Thyroid 2<br>Muscle 1 |
| Tibia | 22 | 22 | 5 | 2 | 3 | 3 (4) | — | — | 1 | — | — | — | — | 1 Scalp |
| Fibula | 4 | 4 | 1 | — | — | — | — | — | — | — | — | — | 1 | — |
| Humerus | 11 | 10 | 3 | 2 | 1 | 1 (4) | 1 | — | — | — | — | 1 | — | — |
| All | 91 | 84<br>92% | 30<br>33% | 10<br>11% | 10<br>11% | 9<br>10% | 4<br>4% | —<br>— | 4<br>4% | —<br>— | —<br>— | 2<br>2% | 2<br>2% | 3<br>3% |

* Figures in parentheses refer to the number of bones involved; other figures to the number of patients.

FIG. 3 —Metastases from osteosarcoma, clinically manifest and found at autopsy; 6 tumours of long bones: 3 of other sites.

TABLE II.—*Osteosarcoma of Long Bones. Primary Sites and Frequency of Clinical Lung Metastases and Extra-pulmonary Metastases (BTR)*

| Primary | Cases | Lung metastases | Extra-pulmonary metastases |
|---|---|---|---|
| Femur | 68 | 48 (70%) | 21 (31%) |
| Tibia | 32 | 22 (68%) | 5 (16%) |
| Fibula | 5 | 4 (80%) | 1 (20%) |
| All leg bones | 105 | 74 (70%) | 27 (26%) |
| Humerus | 19 | 10 (52%) | 3 (16%) |
| All long bones | 124 | 84 (68%) | 30 (24%) |

months for EPM. However, Table IV shows that, though the majority of patients dying from pulmonary metastases never have clinically apparent EPM, yet among those who do LM were noted first in only a third of the cases (10/30). This suggests that a high proportion of clinical EPM are true secondaries; and this is supported by the tendency of osteosarcoma in some bones to metastasize to favoured sites (*e.g.* from distal femur to pelvis). Moreover, the recurrence or persistence of primary tumours, though it has little effect on the incidence of LM, markedly increases the liability to metastases in bone (Fig. 8). None of these observations can be explained on the basis of tertiary deposits in bones from pulmonary secondaries. Nevertheless, the high proportion of EPM found at autopsy (Table III), and most notably those in a range of viscera, including the heart, suggests that tertiary spread does occur sub-terminally.

*Post-metastatic survival*

All 91 patients with clinical metastases died but 11 lived for more than 18 months after their metastases were apparent, and in 8 of these their LM presented after the median time of 5–6 months, Among 42 patients with early metastases. apparent before 5 months (Fig. 4), there were only 4 long survivors (over 24 months), 2 of whom received specific treatment (one lobectomy, one radiotherapy + chemotherapy). The mean total survival time for the other 38 cases with early metastases was only 7·2 months.

Thirty patients with EPM had an average survival of 6·6 months after the EPM were observed. The best prognostic situation appears to be where EPM are confined to lymph nodes. Three such patients survived 7, 33 and

FIG. 4.—Metastases from 91 cases of osteosarcoma of long bones.

TABLE III.—*Osteosarcoma of Long Bones: Anatomical Distribution of Metastases found at Autopsy* (29 *Cases*)

| Primary | Cases | Lung | EPM | Spine | Pelvis | Other bones | Nodes | Heart | Abdomen | Kidney | Adrenal | Liver | Brain | Other sites |
|---|---|---|---|---|---|---|---|---|---|---|---|---|---|---|
| Femur | 18 | 17 | 14 | 4 | 4 | 5 (6)* | 6 | 6 | 7 | 2 | 4 | 1 | 1 | 5 Gall bladder, thyroid, muscle, pancreas 2, chest wall 2, subcutaneous |
| Tibia | 7 | 7 | 7 | — | — | 1 | 1 | 1 | — | — | — | 1 | 2 | 2 Parietal pleura, mediastinum |
| Fibula | 1 | 1 | 1 | — | 1 | — | — | — | 1 | — | — | — | — | — |
| Humerus | 3 | 2 | 2 | 1 | — | — | 1 | 1 | — | 1 | — | — | — | 1 Parietal pleura |
| All | 29 | 27<br>93% | 24<br>83% | 5<br>17% | 5<br>17% | 6<br>21% | 8<br>28% | 8<br>28% | 8<br>28% | 3<br>10% | 4<br>14% | 2<br>7% | 3<br>10% | 8<br>28% |

* Figures in parentheses refer to the number of bones; other figures to the number of patients.

TABLE IV.—*Time Sequence of Metastases and Post-Metastatic Survival from Tumours of Long Bones (BTR)*

| Group | No. | Mean age at onset in years | Mean survival in months: Post LM | Mean survival in months: Post EPM | Primary in distal femur |
|---|---|---|---|---|---|
| A. LM only | 61 | 22·1 | 8·1 | — | 30 (49%) |
| B. LM pre EPM | 10 | 25·5 | 11·1 | 4·2 | 9 (90%) |
| C. LM and EPM synchronous | 7 | 15·9 | 4·4 | 4·4 | 3 (43%) |
| D. LM post EPM | 6 | 14·7 | 8·5 | 14·7 | 3 (50%) |
| E. EPM only | 7 | 29·3 | — | 6·6 | 5 (71%) |

LM Lung metastases.
EPM Extra-pulmonary metastases.

44 months respectively from the time of first treatment of primary tumours of proximal humerus (1) and distal femur (2). These 3 cases were all in Group D of Table IV and largely account for the long survival after EPM in that group. The mean survival post EPM of the other three in Group D was 8·3 months. The post-metastatic survival of 7 patients with EPM only was no better than of those with EPM and LM, but all these 7 had metastases in vertebrae or pelvis or both. The prognosis for a patient with an osseous metastasis is no better than of one with a primary tumour in the same site.

Sixteen patients had clinical EPM in a single site only: 3 cases each in pelvis, vertebrae, other long bones and lymph nodes; 2 in brain, and one each in abdomen and skull. In none, however, would any possible radical surgery for these metastases have been a curative measure as judged by subsequent histories. The somewhat longer duration of life after EPM when these precede LM or appear alone (Table IV, Groups D and E) emphasizes the rapidly fatal effect of lung metastases.

LM (with or without EPM) were recorded for 84 patients. Table V shows the relationship between the time when they were first noted and the mean further survival of patients. This is also shown for both LM and EPM in Fig. 5. Metastases which appear late, although eventually lethal, are more slowly fatal. Patients with over 12 months post-metastatic survival may be grouped as follows:

TABLE V.—*The Behaviour of Lung Metastases from Osteosarcoma of Long Bones (BTR)*

| Month when LM first noted | No. of cases | Survival after lung metastases noted, in months: Range | Survival after lung metastases noted, in months: Mean |
|---|---|---|---|
| 0 | 7 | 3–12 | 6·1±3·1 |
| 1–6 | 41 | 1–18 | 5·0±3·9 |
| 7–12 | 16 | 2–26 | 8·6±7·3 |
| 13–18 | 6 | 5–35 | 10·5±12·0 |
| 19–24 | 5 | 7–25 | 14·6±8·9 |
| Over 24 | 6 | 11–19 | 13·7±2·8 |

3 patients were excluded from this table:
1 with lobectomy at 46 months.
1 with radiotherapy to chest at 60 months.
1 with fatal diabetic coma at 24 months.

Of those with LM under 6 months post treatment—6%.

Of those with LM 6–12 months post treatment—18%.

Of those with LM 12–24 months post treatment—33%.

Of those with LM over 24 months post treatment—50%.

None of the 81 patients in Table V were recorded as having any significant anti-metastatic treatment.

The mean survival also after EPM were noted gradually increased with their later manifestation (Fig. 5, Table VI). It is, however, always less than the mean survival post LM for the same category. This is probably due to the fact that in 16/30 of these patients, LM were present either before or at the same time as EPM, and it is the LM which are usually lethal. Patients with later metastases also tended to have

FIG. 5.—Post-metastatic survival from osteosarcoma of long bones.

a longer pre-diagnostic history of relevant symptoms.

*Late metastases after 24 months from the start of treatment*

Late LM were found in 6 cases of tumour of long bones, with mean survival after metastases of 13·7 months (Table V). One of these patients also had clinical metastases in thyroid and muscle, and others found at autopsy. One patient was a child (M, 13, tumour of distal femur) but late metastases are very rare in children, only 1/46 being recorded here. This case was treated by radiotherapy and delayed amputation at 13 months and metastases appeared at 25 months. Four of the other cases had femoral primaries, all treated by amputation. The sixth was a tumour of proximal fibula, which recurred after radiotherapy and was amputated at 25 months; metastases appeared after a further 55 months. Thus, late LM may occur whatever form of treatment is used and long after complete control of the primary tumour. There were only 2 cases of late EPM, and both already had LM, so the EPM may well have been tertiary. There were no late EPM recorded in children.

In 28 cases of tumours of other bones, late LM were recorded twice, both from relatively favourable sites, namely a tumour of scaphoid of foot, treated by below-knee amputation and a recurrent osteosarcoma of rib. These 2 cases had overall survival of 56 and 83 months respectively. There were no late EPM. There was a third long survivor from tumours of other bones, from a resected osteosarcoma of rib, who lived over 181 months without recurrence or metastases. This the only case which may be considered cured and it is excluded from Table VII, which shows the distribution of metastases from tumours of other bones, and the number of patients—just over half—who died from effects of the primary tumour without clinical metastases. Eighteen of these patients were treated by radiotherapy, 7 by surgical excision (one vertebra, one ilium, 2 mandibles, 3 ribs) and the tumour of scaphoid by amputation. Two patients received no specific treatment.

TABLE VI.—*The Behaviour of Extra-pulmonary Metastases from Osteosarcoma of Long Bones (BTR)*

| Month when EPM first noted | No. of cases | Survival in months after EPM noted | |
|---|---|---|---|
| | | Range | Mean |
| 0 | 1 | — | 3 |
| 1–6 | 10 | 3–7 | $4 \cdot 2 \pm 1 \cdot 7$ |
| 7–12 | 9 | 1–9 | $5 \cdot 2 \pm 3 \cdot 1$ |
| 13–24 | 7 | 2–17 | $7 \cdot 6 \pm 5 \cdot 1$ |
| Over 24 | 2 | 6–12 | $9 \cdot 0 \pm 4 \cdot 2$ |

One case was excluded from this Table, which had metastasis in regional lymph nodes at 3 months, treated by radiotherapy; post EPM survival—41 months.

Time sequences of metastases from tumours of other bones are shown in Fig. 6. The numbers are too small to permit detailed analysis, but the only 2 patients with late metastases mentioned above lived long after these were clinically evident—one with a tumour of rib +30 months, the other with a tumour of pedal scaphoid +26 months. Both had specific local treatment for their metastases—the rib case with orthovoltage therapy, the other with thoracotomy. In the other 11 patients, survival after clinical metastases averaged only 5 months (range 2–8 months). This is obviously a heterogeneous group, and the scaphoid tumour behaved more like a long bone osteosarcoma of a distal site. There were only 3 children in this group.

With an average survival of only 7 months, the proportion of cases with clinical LM (41%) is similar to those of the long bone cases who develop LM in that time (57/124 = 46%). Paradoxically, if the life of these patients could be extended, the proportion showing metastases would increase and might equal or exceed that for the long bone tumours, as the presence of an uncontrolled primary implies the continuation of metastatic risk. Until there is complete control of the primary tumour the later treatment of osteosarcoma of calvarium, spine or the limb girdles is virtually only palliative. It has been reported by several authors that osteosarcoma of mandible has a better than average prognosis, but in this study 4 out of 5 cases died without clinical metastases; of autopsy cases 2/2 had LM and 1/2 had EPM (Tables VII and VIII).

*Metastases found at autopsy* (Tables III and VIII)

Twenty-seven of 29 patients with tumours of long bones had pulmonary metastases: in only 2 of these were the LM solitary, in each case from a femoral primary. All 10 cases with LM from tumours of other bones had multiple LM.

Figure 3 shows the metastases recorded

FIG. 6.—Metastases from 13 cases of osteosarcoma of other than long bones.

TABLE VII.—*Anatomical Distribution of Clinical Metastases from Osteosarcoma of Other Than Long Bones (BTR)*

(27 cases with clinical metastases or dead from effects of primary)

| Primary | Cases | No clinical metastases | Lungs | EPM | Spine | Other bones | Other sites |
|---|---|---|---|---|---|---|---|
| Pelvis | 12 | 5 | 7 | — | — | — | — |
| Sacrum | 2 | 1 | 1 | — | — | — | — |
| Spine | 3 | 3 | — | — | — | — | — |
| Scapula | 1 | — | — | 1 | 1 | — | — |
| Ribs | 2 | — | 2 | 1 | 1 | 1 | — |
| Jaw | 5 | 4 | (One case reported to have generalized metastases) | | | | |
| Skull | 1 | 1 | — | — | — | — | — |
| Scaphoid | 1 | — | 1 | — | — | — | — |
| All | 27 | 14<br>52% | 11<br>41% | 2<br>7% | 2<br>7% | 1<br>4% | —<br>— |

in 9 BTR patients for whom both clinical and autopsy records are available. The majority of LM were clinically evident; in other viscera the predominance of terminal events is notable. Similarly, comparing Tables I and III, VII and VIII, whereas the incidence of LM is not markedly greater in the autopsy cases, that of EPM is greatly increased, particularly in the viscera, with metastases in lymph nodes, heart and abdomen each being found in about a quarter of autopsies.

*Heart metastases* were not recorded clinically in any of the 152 BTR cases, but 9 examples were recorded (21%) in 43 autopsies. All but one were derived from long bone tumours—6 femora, one tibia, one humerus, one vertebra. Except in one patient, the lungs were also involved: 7 with multiple LM and one with a solitary metastasis in the right lung. Cardiac metastasis appears to be a relatively late phenomenon and is recorded in only one of the comparatively shortlived cases of tumours of other bones (Table VIII). A patent foramen ovale was noted only once, in a patient who had only heart and lung metastases. The right side of the heart was involved more often than the left (6R : 3L), and metastases were related about equally to endocardium and epicardium. In only 3 of these 9 cases was the metastasis described in the myocardium.

*Lymph nodes* were invaded by tumour in 11/43 cases (26%), with similar frequency from tumours of long bones and other bones. In only 4, however, was involvement of the regional nodes reported—the sites most frequently recorded were hilar, mediastinal, mesenteric and abdominal nodes.

*Abdominal metastases* were found in 9/43 cases (21%), with a preponderance from tumours of long bones. This is almost certainly the result of haemic spread. It is frequently associated with metastases of liver, kidney, gall bladder or pancreas.

*The frequency of osseous metastases* is well known and is shown in Tables I, III, VII and VIII. They appear with similar frequency from tumours of the 3 major long bones and slightly less often from those of axial and girdle bones. The vertebrae and pelvis are by far the most common metastatic sites, particularly from osteosarcoma of femur. At autopsy 14/29 tumours of long bones (48%) and 5/14 of other bones (36%) had osseous metastases; they are exceeded in number only by LM. There were 3 examples of bone metastases distal to the elbow and knee joints—one in the ulna from a tumour of distal femur, and metastases in tibia and talus from a primary of proximal humerus. Other less common sites found clinically were femur and humerus (3 each), skull (2), and one each in mandible, mastoid, rib, sternum and sacrum. Osseous metastases are clinically

TABLE VIII.—*Autopsy Records of Osteosarcoma of Other Than Long Bones. Anatomical Distribution of Metastases in* 14 *Cases with Death Due to Tumour*

| Primary | Cases | D. from primary | No. of secondaries | Lungs | EPM | Spine | Other bones | Nodes | Heart | Abdomen | Kidney | Adrenal | Liver | Other sites |
|---|---|---|---|---|---|---|---|---|---|---|---|---|---|---|
| Pelvis | 7 | 2 | 1 | 6 | 4 | 2 | — | 2 | — | — | 1 | 1 | — | 2 Pleura 2 |
| Vertebra | 4 | 3 | 2 | 2 | 2 | — | 1 | 1 | 1 | 1 | — | — | 1 | 2 Thyroid, subcutaneous, muscles, gall bladder, spleen and pancreas |
| Jaw | 2 | 1 | — | 2 | 1 | — | 1 | — | — | — | — | — | — | 1 Chest wall |
| Skull | 1 | 1 | — | — | 1 | — | 1 | — | — | — | — | — | — | — |
| All | 14 | 7<br>50% | 3<br>21% | 10<br>71% | 8<br>57% | 2<br>14% | 3<br>21% | 3<br>21% | 1<br>7% | 1<br>7% | 1<br>7% | 1<br>7% | 1<br>7% | 5 cases<br>36% |

more frequent when there has been local recurrence of the primary (Fig. 8).

Table VIII is an analysis of metastatic sites recorded for 14 autopsied cases of osteosarcoma of other bones. Three (21%) had no macroscopic metastases. LM were noted in 10 (71%) and osseous metastases in 5 (35%). The general pattern of EPM resembles that of the long bone tumours but at a lower level throughout. Osseous metastases were found only in skull, ribs and vertebrae. One patient, not included in Table VIII, died from a tumour embolism of the right subclavian vein, right auricle and pulmonary artery, post-operative after scapulectomy, but without other metastases.

*Ultimate causes of death from osteosarcoma*

Most patients die at home, without adequate medical records, and the following information is derived from autopsy reports. As with many forms of cancer, the commonest terminal factor is " malignant cachexia "—emaciation and asthenia, with or without anaemia, with multiple LM and EPM and often a large uncontrolled primary tumour. About 90% have multiple bilateral LM which are sometimes immense. Rather less than half have pleural effusions with some degree of lung collapse, this being commoner than bronchopneumonia or bronchitis. About a third have metastatic or thrombotic/embolic cardiovascular disease and about a quarter may have obstruction or infection of the urinary tract. Other less frequent causes of death are haemorrhage into a lung or pleural cavity, septicaemia, tracheal obstruction from tumour metastatic in the thyroid and intestinal obstruction due to abdominal secondaries.

*Sex and age distribution and metastases*

The figures given in Table IX show no significant differences between the sexes for metastases either from tumours

TABLE IX.—*Sex Frequencies of Metastases from Osteosarcoma (BTR)*

| Tumours of long bones | 74 Males | 50 Females |
|---|---|---|
| Only lung metastases | 34 (46%) | 27 (54%) |
| Only extra-pulmonary metastases | 3 (4%) | 4 (8%) |
| All lung metastases (±EPM) | 51 (66%) | 33 (66%) |
| All EPM (±LM) | 20 (27%) | 10 (20%) |
| Tumours of other bones | 15 Males | 13 Females |
| All metastases (LM and EPM) | 6 (40%) | 7 (54%) |

of long bones or of other bones. Age again has little effect, at least with tumours of long bones: Of 42 children under 15 years, 28 had LM = 67%; 10 had EPM = 24%. Of 82 adults over 15 years, 56 had LM = 68%; 20 had EPM = 24%. However, in children EPM were almost invariably in bone, only 3 exceptions being recorded—one each in liver, lymph nodes and abdomen. Late EPM, preceded by LM, occurred in 2 adults; there were no late EPM in children and only one late LM, but 5 late LM in adults. There were only 3 children with tumours of other bones; one had LM; none had EPM.

*Treatment of the primary tumour and metastases*

Table X gives comparative figures for metastases from long bone tumours in the 3 main treatment groups. There are no statistically significant differences.

*The incidence and effect of local recurrence*

Local recurrence occurred in 35 of 124 long bone sarcomata (28%). This includes one radiotherapy case, treated by the " Cade " method (Lee and Mackenzie, 1964) where viable tumour was found in the amputation specimen though there was no clinical recurrence, before or later, and 2 patients with stump recurrence following transfemoral amputation after radiotherapy.

The average time for recurrence was 5·5 months from starting treatment (range

TABLE X.—*Treatment of Primary Osteosarcoma of Long Bones and Incidence of Metastases (BTR)*

| Treatment group | Cases | Lung metastases | Extra-pulmonary metastases | Dead from tumour: With secondaries | Dead from tumour: Without secondaries |
|---|---|---|---|---|---|
| A1. Surgery | 50 (54–4*) | 34 (68%) | 14 (28%) | 38 (76%) | — |
| A2. "Cade" method | 48 (50–2*) | 34 (71%) | 11 (23%) | 37 (77%) | 4 (8%) |
| A3. Combined RT and surgery | 20 | 16 (80%) | 6 (30%) | 17 (85%) | 1 (5%) |

* Cases with insufficient information.

FIG. 7.—Recurrence of primary osteosarcoma and metastases.

2–20 months), which is similar to the median for LM and slightly longer than the mean recurrence time of 3·7 months reported by Jenkin (1973). Only 2 local recurrences occurred after 12 months.

Figures 7 and 8 compare the frequency of metastases in 28 cases with local recurrence and 82 without (7 patients with and 5 without recurrence died with no recorded metastases, and there were 2, not included in either group, where the primary showed no response to treatment). There are no marked differences between the 2 groups for the first 6 months; after this Fig. 7 shows an increase in metastatic frequency related to local recurrence, and a second rise after 18 months. Figure 8 shows that the difference is mainly due to a marked increase in bone metastases from tumours with recurrence.

FIG. 8.—Recurrence of primary osteosarcoma and metastases to lung and bone.

This is statistically significant at 24 months ($P < 0{\cdot}05$).

It would seem that nearly all metastases which are clinically manifest within 6 months of starting treatment are already seeded at that time. This includes most lung metastases but only a small proportion of osseous metastases, and there is prolonged dissemination so long as active tumour remains in the body.

Local recurrence occurred in

18/68 tumours of femur (26%)
5/32 tumours of tibia (16%)
3/5 tumours of fibula (60%)
9/19 tumours of humerus (47%)

(One tumour of fibula recurred after local excision, was treated by radiotherapy and again recurred; it was then amputated.)

Only the difference between humerus and tibia is statistically significant ($P < 0{\cdot}02$) but the greater frequency of local recurrence in the humerus may be attributed to the larger proportion treated by radiotherapy, which has a positive correlation with recurrence:

Humerus, radiotherapy (±amputation (16/19 (84%); recurrence 47%.

Femur, radiotherapy (±amputation) 40/68 (58%); recurrence 26%.

Tibia, radiotherapy (±amputation) 11/32 (31%); recurrence 16%.

It is peculiar that the humerus, with a high local recurrence rate, has the lowest rate of EPM, but those which do occur are almost entirely osseous, thus following the trend shown in Fig. 8. Of 35 patients with recurrence, the treatment of the primary was:

| | |
|---|---|
| Radiotherapy | 17 (48%) |
| Radiotherapy + limb ablation | 13* (37%) |
| Amputation | 4 (12%) |
| Local excision | 1 only (3%) |

* 10 of these 13 had limb ablation for tumour recurrence. In addition, the tumour of fibula with recurrence after excision was then treated by radiotherapy and later amputated for further tumour activity.

TABLE XI.—*Dosage of Irradiation to Tumours of Long Bones and Local Recurrence (BTR)*

| Orthovoltage | | | | | Megavoltage | | | | |
|---|---|---|---|---|---|---|---|---|---|
| Dose (roentgen) | Period of treat-ment (days) | Cases | Immediate ablation | Local recurrence | Dose (rad) | Period of treat-ment (days) | Cases | Immediate ablation | Local recurrence |
| <3000 | 3–21 | 6 | 6 | — | <3000 | 4–55 | 4 | 3 | 1 |
| 3000 | 12–34 | 3 | 2 | 1 | 3000 | 11-60 | 5 | 1 | 3 |
| 4000 | 23–98 | 6 | 2 | 4 | 4000 | 22–44 | 4 | — | 3 |
| 5000 | 14–94 | 3 | — | 3 | 5000 | 36–44 | 6 | — | 3 |
| 6000 | 53–86 | 2 | — | 1 | 6000 | 30–63 | 12 | — | 6 |
| 7000 | 17 | 1 | — | — | 7000 | 38–75 | 6 | 1 | 3 |
| 8000 and over | 95–120 | 2 | — | 1 | 8000 and over | 48-79 | 4 | — | 2 |
| All | | 23 | 10 | 10 (43%) | All | | 41 | 5 | 21 (51%) |
| All over 5000 | | 8 | — | 5 (62%) | All over 5000 | | 28 | 1 | 14 (50%) |

2 cases omitted who were given palliative irradiation of under 1000 roentgen (OVT).
4 cases omitted where dosage could not be ascertained.

Of 54 patients treated by surgery, 5 had local recurrence (9%).

Of 50 + 20 treated by radiotherapy ± surgery, 30 had recurrence (43%).

Of the 30 cases of recurrence after radiotherapy, 10 occurred among 23 who were treated by orthovoltage, and 20 among 40 given megavoltage therapy. The fibula cited above was also treated by megavoltage, making a final figure of 21 out of 41 cases. Table XI shows the dosage given and numbers with local recurrence for 64 cases of long bone tumours treated by radiotherapy. Important variables are not only the quality and total dosage of irradiation, but also field sizes, the number of treatment fractions and duration of therapy. The scope of this paper does not permit a detailed analysis, but it is emphasized that regimens used at various treatment centres varied widely. Of 28 patients treated with less than 5000 rad or 5000 R, 14 had immediate ablation of the tumour-bearing limb; one of these patients had a stump recurrence after amputation for recurrent tumour growth. Megavoltage therapy does not appear markedly better than orthovoltage in controlling the primary tumour, and local recurrence may occur even with maximum dosage of irradiation.

The overall effect of local recurrence on the 5-year survival rate is small, but contributes to the poorer results of cases treated by radiotherapy (Price and Jeffree, 1973). 33/35 patients with recurrence have died (94%), with a mean survival of those 33 of 17·7 months. 68/87 cases without local recurrence (78%) died of the effects of tumour (one other patient died after 146 months with no residual tumour) with a mean survival of 18·8 months. There is little difference in survival times of those who died, the lethal factor being LM in the great majority of cases, *but all except 2 of the cures were in cases with no local recurrence.*

With osteosarcoma of other bones, the problem is not so much that of recurrence as of complete inability to control the primary tumour. Of 27 deaths from tumour (there was one long survivor), 14 had no clinical metastases; death was due to local effects of the primary (Table VII). A similar result is shown in the autopsy records of 14 patients in Table VIII. The short survival of these cases—mean 7 months—

does not permit any further useful analysis.

### DISCUSSION

*Metastatic frequency*

The inevitable appearance of lung metastases in the majority of cases is well known, but perhaps the almost equal terminal incidence of EPM is not so widely appreciated, nor their attendant distress to the patient, yet most patients dying with LM will also have EPM (Table III).

Since we know neither the duration of tumour growth before diagnosis nor the precise time of metastatic spread, one cannot be certain of the significance of time sequences of LM and EPM in any particular patient. At the time of diagnosis and initiation of treatment there is no sure method of deciding which tumour will metastasize—when and where. The mitotic activity may be some guide, but its evaluation is open to technical and sampling errors, and the most important prognostic factor is still the extent of our ability to control the primary tumour, which depends mainly upon its site. It must be emphasized that any anti-metastatic treatment, to be effective, must deal with the whole body in every case, even though the lung metastases are of paramount importance.

*The time factor* is important in understanding several aspects of tumour behaviour. When survival is longer than the median time for LM (5–6 months), clinical metastases may present which would not otherwise be evident. Nearly half the patients with tumours of other bones succumb within 6 months to local effects of tumour growth, with no overt metastases.

As already shown by survival curves (Price and Jeffree, 1973), after 2 years metastatic frequency diminishes and individual prognosis improves.

With the median time for LM at 5–6 months, the " Cade " method alone can never cure more than 50% of patients. All published series agree on this time for LM, whether treatment has been " Cade " or ablation.

After the median time for LM, metastases are less rapidly lethal, probably owing to their slower growth, which may reflect enhanced host resistance (Table V). This less aggressive behaviour of later metastases has been reported for other cancers—breast, thyroid, kidney and seminoma. If it is due to a host-immune reaction, this must be systemic and not localized to any particular organ or type of tissue, since it holds good not only for LM but for EPM in a number of different sites.

*Metastases in special sites*

*Other bones.*—As with metastatic carcinoma, vertebrae and pelvis are most frequently involved. Osseous metastases were reported clinically or radiographically for 18% of the long bone tumours, closely conforming with the 14% of McKenna *et al.* (1966). Lockshin and Higgins (1966) found 41% with bone metastases among 22 patients dying in hospital who were examined terminally by radiography or autopsy. This agrees well with the 48% reported here with osseous metastases at autopsy. Owing to their shorter average survival, patients with primary tumours of other bones show fewer osseous metastases either clinically (7%) or at autopsy (36%).

*Heart metastases.*—These are subterminal events, being found in our series only at autopsy; if they are secondaries, they must grow more slowly than metastases in other sites. Yet in only 2 of 9 cases did they appear to be tertiary from lung secondaries, in the other 7 they may have been true secondaries or tertiary from large metastases in, for example, the pelvis. Autopsy records suggest that, even with advanced modern surgical techniques, it would seldom, if ever, be possible to remove a heart metastasis with any good hope of success. Possibly their late effect upon cardiac function may rarely be misinterpreted as drug cardiotoxicity.

*Cerebral metastases.*—These were recorded at autopsy for 3 long bone tumours (one femur, 2 tibia), the sites involved being respectively surface of temporal lobe, cerebellum and left frontal lobe; 6 separate unspecified sites. Two patients also had multiple LM. Surgical removal of brain metastases may deserve careful consideration in the absence of clinical metastases elsewhere.

*Muscle.*—As with other cancers, the infrequency of muscular metastases (even in the heart) is noteworthy. Yet the wide spectrum of metastases suggests that tumour cells must also reach muscle, but find the environment unsuitable for further growth. Why this is so is not known but it surely merits further careful experimental investigation. The spleen likewise is seldom involved.

*Lymph node metastases.*—These were found in 26% of all autopsies, but metastases in regional nodes in only 10% of the 29 autopsied cases of tumours of long bones, and in only 3% of the 124 clinical records. These figures may be compared with Schwinn and McKenna's (1973) report of 6% of lymph node metastases in amputation specimens. Caceres, Zaharia and Tantalean (1969) found 11·4% with lymphoid secondaries among 35 cases. None of these figures approach the 50% frequency of lymph node invasion asserted by Makai, Belan and Malek (1971) on lymphographic evidence.

Age and sex have little effect upon metastatic frequency or distribution.

Local recurrence contributes very little to the frequency of lung metastases (Fig. 8) since most of these are probably seeded before treatment is begun; a supposition supported by the frequency of early clinical LM. It is presumably just this fact that makes the " Cade " technique an acceptable compromise, otherwise one might anticipate a much worse comparison with other methods than is found (Price and Jeffree, 1973; Trifaud and Meary, 1972). By contrast, the clinical appearance of osseous metastases is significantly increased by local tumour recurrence, suggesting that only about one-third of these have been seeded at the time of first treatment. Similarly in Ewing's sarcoma, which metastasizes freely to other bones, local recurrence is often associated with further metastatic spread (Macintosh, Price and Jeffree, 1975).

*Longer post-metastatic survival after later metastases* (Fig. 5)

This relationship agrees with the similar findings of Joseph, Morton and Adkins (1971) who noted positive correlations between the time of onset of metastases, the estimated tumour doubling time, and length of survival.

*Mode of spread*

The general pattern found in both clinical and autopsy studies supports the present view that the venous pathway of tumour dissemination is usual. Bearing in mind that in addition to the caval vessels there is a wide network of the vertebral vein system (Batson, 1957) in which reversal of blood flow may take place, it is easy to comprehend how limb tumours may spread to the axial and girdle bones and elsewhere. There is some indication that leg tumours tend to metastasize below the diaphragm and arm tumours above it, and that osteosarcoma of humerus is less likely to metastasize than that of femur, but secondaries from either limb may appear almost anywhere. Osteosarcoma of other bones conforms to the long bone pattern apart from the short time available in most cases for the spread and growth of metastases.

*The strategy of therapy*

To what extent can this study assist in planning treatment?

1. All cases of osteosarcoma must be regarded as systemic disease, the majority of tumours having already spread beyond the primary site at the time of diagnosis. Lung metastases in particular must usually be seeded by this time, though they may occasionally lie dormant for over 5 years after successful amputation as in 3 BTR cases.

2. Although lung metastases are the lethal factor for most patients, effective anti-metastatic treatment should include the whole body. Maybe it will be necessary to employ specific intrathecal treatment for the central nervous system.

3. Tumour treatment resolves into four stages: (I) Complete control of the primary and " prophylactic " treatment for occult micrometastases; (II) treatment of first metastases, including regional lymph nodes and early local recurrence. Lymphangiography might well be used as well as chest radiography, and isotope scanning of at least vertebrae and pelvis should be more often employed; (III) prevention of late metastases (over 2 years) and (IV) treatment of advanced multifocal disease.

At present most cases progress through stages I, II and IV, but half the patients with osteosarcoma of other bones die in stage I with uncontrolled primaries.

4. Later-appearing metastases, both LM and EPM, are more slowly lethal, so merit careful assessment and active therapy—surgery, radiotherapy and chemotherapy, or possibly all three. Estimation of the tumour doubling time from serial chest radiographs, as advocated by Joseph *et al.* (1971) may be valuable in the selection of cases suitable for thoracotomy.

5. If this feature of later metastases is related to enhanced resistance, this might be the critical time when immunotherapy should be practised—not when there is a large mass of actively growing tumour tissue.

6. The history of one patient (BTR 3210, F. 9, osteosarcoma of humerus) who engaged in active sports after a forequarter amputation, suggests that this physical exertion may have been partly responsible for her unusually widespread metastases. Patients should be advised to avoid regular forceful exertion for at least 3 years.

The authors emphasize their appreciation of the prolonged interest and support of numerous colleagues who by regularly referring their cases have contributed to this present series of osteosarcomata. Moreover, to laboratory workers such a study would have been impossible without the guidance obtained by discussion of clinical and radiological problems with those responsible for these aspects of the care of patients.

Likewise, thanks are due to all the pathologists who responded to the request for copies of osteosarcoma autopsy reports, particularly to those whose postmortem findings have been used in this study: Drs G. M. G. Barton, A. H. Cameron and J. Cocker, Professors I. Doniach and D. L. Gardner, Dr G. J. Hardy, Professors T. F. Hewer, D. H. Mackenzie, Drs J. S. McKinnell, G. Meachin, A. I. D. Prentice, A. H. T. Robb-Smith, G. A. C. Summers, the late Dr A. L. Taylor and Dr R. F. Welch. Further autopsy data from the files of the London Bone Tumour Panel were included by kind permission of the members. Mrs J. E. Nutt rendered invaluable services in the collection of clinical records and radiographs also with extensive correspondence. The Bristol Bone Tumour Registry and this work are supported by generous grants from the Cancer Campaign for Research.

### REFERENCES

Batson, O. V. (1957) The Vertebral Vein System. *Am. J. Roentgen.*, **78**, 195.

Caceres, E., Zaharia, M. & Tantalean, E. (1969) Lymph Node Metastases in Osteogenic Sarcoma. *Surgery, St Louis*, **65**, 412.

Jenkin, R. D. T. (1973) The Management of Osteosarcoma and Ewing's Sarcoma. The Colston Papers No XXIV. Ed. C. H. G. Price and F. G. M. Ross. London: Butterworths. p. 229.

Joseph, W. L., Morton, D. L. & Adkins, P. C. (1971) Prognostic Significance of Tumor Doubling Time in Evaluating Operability in Pulmonary Metastatic Disease. *J. thorac. cardiovasc. Surg.*, **61**, 23.

LEE, E. S. & MACKENZIE, D. H. (1964) Osteosarcoma. A Study of the Value of Preoperative Megavoltage Radiotherapy. *Br. J. Surg.*, **51**, 252.

LOCKSHIN, M. D. & HIGGINS, I. T. T. (1966) Bone Metastasis in Osteogenic Sarcoma. *Archs intern. Med.*, **118**, 203.

MACINTOSH, D. J., PRICE, C. H. G. & JEFFREE, G. M. (1975) Ewing's Tumour: A Study of the Behaviour and Treatment of 47 Cases. *J. Bone Jt Surg.* In the press.

MAKAI, F., BELAN, A. & MALEK, P. (1971) Lymphatic Metastases of Bone Tumors. *Lymphology*, **3**, 109.

McKENNA, R. J., SCHWINN, C. P., SOONG, K. Y. & HIGINBOTHAM, N. L. (1966) Sarcomata of the Osteogenic Series (Osteosarcoma, Fibrosarcoma, Chondrosarcoma, Parosteal Osteogenic Sarcoma and Sarcomata arising in Abnormal Bone). Analysis of 552 Cases. *J. Bone Jt Surg.*, **48A**, 1.

PRICE, C. H. G. & JEFFREE, G. M. (1973) Metastatic Spread of Osteosarcoma. *Br. J. Cancer*, **28**, 515.

SCHWINN, G. P. & McKENNA, R. J. (1973) The Biologic Behavior of Osteosarcoma. *Proc. 7th Natn. Cancer Conf.* Philadelphia: Lippincott. p. 925.

TRIFAUD, A. & MEARY, R. (1972) *Prognostic et Traitement des Sarcomes Ostéogéniques.* Paris: Masson. p. 123.